# Nicotinic Amidoxime Derivate BGP-15, Topical Dosage Formulation and Anti-Inflammatory Effect

**DOI:** 10.3390/pharmaceutics13122037

**Published:** 2021-11-29

**Authors:** Ágota Pető, Dóra Kósa, Ádám Haimhoffer, Pálma Fehér, Zoltán Ujhelyi, Dávid Sinka, Ferenc Fenyvesi, Judit Váradi, Miklós Vecsernyés, Alexandra Gyöngyösi, István Lekli, Péter Szentesi, Annamária Marton, Imre Gombos, Barbara Dukic, László Vígh, Ildikó Bácskay

**Affiliations:** 1 Department of Pharmaceutical Technology, Faculty of Pharmacy, University of Debrecen, Nagyerdei Körút 98, H-4032 Debrecen, Hungary; peto.agota@pharm.unideb.hu (Á.P.); kosa.dora@pharm.unideb.hu (D.K.); haimhoffer.adam@pharm.unideb.hu (Á.H.); feher.palma@pharm.unideb.hu (P.F.); ujhelyi.zoltan@pharm.unideb.hu (Z.U.); sinka.david@pharm.unideb.hu (D.S.); fenyvesi.ferenc@pharm.unideb.hu (F.F.); varadi.judit@pharm.unideb.hu (J.V.); vecsernyes.miklos@pharm.unideb.hu (M.V.); 2 Doctoral School of Pharmaceutical Sciences, University of Debrecen, Nagyerdei Körút 98, H-4032 Debrecen, Hungary; 3 Institute of Healthcare Industry, University of Debrecen, Nagyerdei Körút 98, H-4032 Debrecen, Hungary; 4 Department of Pharmacology, Faculty of Pharmacy, University of Debrecen, Nagyerdei Körút 98, H-4032 Debrecen, Hungary; gyongyosi.alexandra@pharm.unideb.hu (A.G.); lekli.istvan@pharm.unideb.hu (I.L.); 5 Department of Physiology, Faculty of Medicine, University of Debrecen, Nagyerdei Körút 98, H-4032 Debrecen, Hungary; szentesi.peter@med.unideb.hu; 6 Institute of Biochemistry, Biological Research Center, Temesvári Körút 62, H-6726 Szeged, Hungary; vigh@brc.hu (L.V.); marton.annamaria@brc.hu (A.M.); gombos.imre@brc.hu (I.G.); dukic.barbara@brc.hu (B.D.)

**Keywords:** nicotinic amidoxime derivate, BGP-15, ointments, surfactants, anti-inflammatory drug, drug delivery, dosage formulation, antioxidant, PARP inhibitor

## Abstract

BGP-15 is a Hungarian-developed drug candidate with numerous beneficial effects. Its potential anti-inflammatory effect is a common assumption, but it has not been investigated in topical formulations yet. The aim of our study was to formulate 10% BGP-15 creams with different penetration enhancers to ensure good drug delivery, improve bioavailability of the drug and investigate the potential anti-inflammatory effect of BGP-15 creams in vivo. Since the exact mechanism of the effect is still unknown, the antioxidant effect (tested with UVB radiation) and the ability of BGP-15 to decrease macrophage activation were evaluated. Biocompatibility investigations were carried out on HaCaT cells to make sure that the formulations and the selected excipients can be safely used. Dosage form studies were also completed with texture analysis and in vitro release with Franz diffusion chamber apparatus. Our results show that the ointments were able to reduce the extent of local inflammation in mice, but the exact mechanism of the effect remains unknown since BGP-15 did not show any antioxidant effect, nor was it able to decrease LPS-induced macrophage activation. Our results support the hypothesis that BGP-15 has a potential anti-inflammatory effect, even if it is topically applied, but the mechanism of the effect remains unclear and requires further pharmacological studies.

## 1. Introduction

BGP-15 is a nicotinic amidoxime derivative, a Hungarian-developed drug candidate, which was originally intended to alleviate neuro-, nephro- and myelotoxic effects of different cytostatic preparations [1,2,3,4,5]. In the past few years, several other beneficial effects of the drug have been revealed, including the insulin sensitizing effect [1,6,7,8,9], and some scientific publications also mention its potential anti-inflammatory effect [5,10]. BGP-15 has entered into clinical phase II in the indication of diabetes [1,7,8], but the determination of the proper indication is still ongoing as we still have little knowledge about the drug. As for this matter, the exact mechanism of the effect is still unknown, though many research groups are studying BGP-15. In our previous work, we have already summarized those effects and pharmacological properties of the drug, which have been revealed in the past [11]. As mentioned above, the potential anti-inflammatory effect is attributed to the drug, but it is an under investigated field. Topical application of the drug is also a less investigated research area, as well as the external anti-inflammatory effect of BGP-15. Farkas et al. have formulated creams of BGP-15, though the indication was slightly different. They investigated the photoprotective effect of the ointments by pretreating mice with the preparations, then exposed the animals to direct UV radiation. They found that the creams reduced the number of sunburn cells significantly and they observed the downregulation of epidermal cytokines IL-10 and TNF-α [12]. Considering the abovementioned properties, investigating the possible anti-inflammatory effect of BGP-15 in external dosage forms could complete our current knowledge and provide further useful information about the drug.

Drugs can be administered into the human body by numerous anatomical routes. They can be intended for systemic effects or directed to a specific organ. The choice of the right route of administration depends on the indication field and the desired effect. The oral route of drug administration is the most popular and most conventional method. It is easy and highly accepted among patients, however, there are some limitations, such as gastrointestinal decomposition of the drug, irritation or difficulty swallowing. Parenteral administration is another frequent route, which avoids the gastrointestinal tract, the most frequently used invasive method of drug administration. The main disadvantage of parenteral administration is the poor patient compliance. Transdermal or topical application of drugs is a favorable route because it does not require the presence of a trained person, no pain is associated with it, it avoids the harsh conditions of the gastrointestinal tract and allows local treatment of some diseases [13,14].

The anti-inflammatory effect of topical preparations can be improved by selecting the right vehicle and the right excipients for the formulation. There are common penetration enhancers, combinations thereof which are preferably used in ointments because they can enhance the effect, by ensuring good drug delivery [15]. Propylene glycol (PG) and isopropyl myristate (IPM) can synergistically enhance the penetration of the active substance (e.g., diclofenac sodium) from gels, and, thus, potentiate the anti-inflammatory effect [16]. The combination of sucrose esters and Transcutol also positively affects the permeation of ibuprofen into deeper skin layers, thus enhancing its effect [17]. Suthasinee et al. studied the anti-inflammatory effect of different creams containing Curcuma mangga extract, their choice of emulsifiers was Cremophor A6; A25; glyceryl monostearate in each preparation to achieve proper bioavailability [18]. Penetration of piroxicam according to d’Arpino et al. can be greatly improved with the combination of PG, petrolatum and Transcutol P compared to other vehicles they have studied [19]. Kataras et el. also investigated piroxicam, and how to improve the release rate and solubility of the drug. They found that the combination of Labrasol and Gelucire 44/14 has a positive effect on drug release [20].

**Figure 1:**
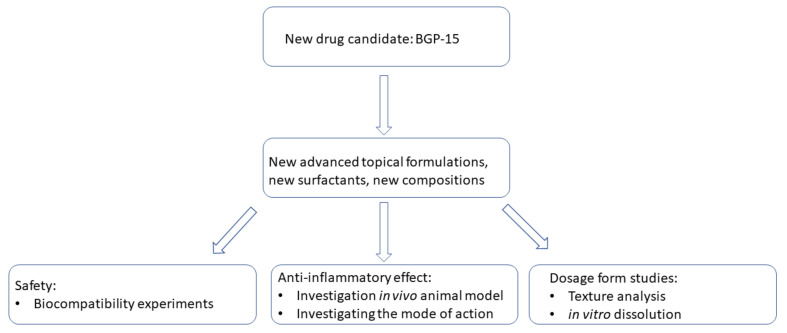
Overview of the study design and formulation strategy.

The objective of our study (Figure 1) was to formulate o/v emulsion ointments of BGP-15 with different surfactants (sucrose esters, Labrasol, Cremophor A6; A25) and incorporate penetration enhancers into the compositions to ensure good drug delivery and improve the bioavailability and stability of the active substance. With these ointments our aim was to test the anti-inflammatory effect of BGP-15 in vivo and perform dosage form studies as well: in vitro release with the help of Franz diffusion chamber apparatus, texture analyzing studies and biocompatibility investigations to check in vitro toxicity. Since the exact mechanism of effect is still unknown of BGP-15, we also aimed to evaluate the antioxidant effect and ability of BGP-15 to decrease macrophage activation.

## 2. Materials and Methods

### 2.1. Materials

SP50, SP70 and PS750 sucrose esters were kindly gifted by Sisterna (Roosendaalc, The Netherlands). 3-(4,5-Dimethylthiazol-2-yl)-2,5-diphenyltetrazolium bromide (MTT paint), Dulbecco’s Modified Eagle’s Medium (DMEM), phosphate buffered saline (PBS), Trypsin-EDTA, heat-inactivated fetal bovine serum (FBS), l-glutamine, non-essential amino acids solution, penicillin–streptomycin, allyl isothiocyanate (AITC), LPS were purchased from Sigma Aldrich (St. Gallen, Switzerland). We purchased 96-well plates, culturing flasks from Corning (Corning, New York, NY, USA). Cetostearyl alcohol, propylene glycol, stearic acid, isopropyl myristate, conservant solution were obtained from Hungaropharma Ltd. (Budapest, Hungary). HaCaT cells were supplied from Cell Lines Service (CLS, Heidelberg, Germany). BGP-15 was purchased from SONEAS Chemicals Ltd. (formerly known as Ubichem Pharma Services) Illatos street 33, Budapest, H-1097, Hungary. Cremophor A6, A25 was purchased from BASF (Ludwigshafen, Germany). Transcutol was a kind gift from Gattefossé (Lyon, France). Firefly luciferase substrate was purchased from Promega (Madison, WI, USA).

### 2.2. Animals

Our experiments were carried out on 25–35 g, 3–6 months old C57BL/6J mice kept under adequate pathogen-free circumstances at 24–25 °C. They were fed with standard rodent chow and had access to water ad libitum. Light–dark cycle was 12:12 h. The study was approved by the local Ethics Committee of University of Debrecen under the number HB/15-ÉLB/1921-6/2020 (date: 7 September 2020; date of expiry: 7 September 2025). The experiment was designed to minimize suffering and the number of animals.

### 2.3. Formulation of Ointments

Different surfactants were incorporated into the formulations: sucrose esters (SP50, SP70, PS750), Cremophor A6:A25, Labrasol (Table 1). The ointments were produced by melting stearic acid, cetostearyl alcohol, isopropyl myristate (IPM) and mixed to prepare the oily phase of the formulation. The aqueous phase containing propylene glycol (PG), surfactant, glycerol, purified water was heated to the same temperature as the oily phase (~60 °C), mixed together and cooled down to room temperature. After that, BGP-15 solution (10%) and conservant solution was added to the preparation [21].

### 2.4. Texture Analyzing Studies

Resistance of the ointments was evaluated with the help of CT3 Texture Analyzer (Brookfield, Middleboro, MA, USA). During the experiment compression test was performed in normal mode with the following settings: target value (10 mm), target load (4 g), target speed (0.5 mm/s) [22].

### 2.5. In Vitro Release

In vitro release of BGP-15 from the ointments was performed with the help of Franz diffusion chamber apparatus. During the experiment a membrane is placed between the donor and the acceptor phase. The concentration profile of the test substance is obtained by taking samples at predetermined times. Samples of 300 mg were placed on artificial cellulose acetate membrane (0.45 µm pore size) as donor phase, as receptor phase pH = 5 buffer was chosen because of the active ingredient’s good water solubility (28 mg/mL in deionized water at 25 °C). The membrane was pre-treated with isopropyl myristate to characterize the lipophilic property of the skin. The rotation of the magnetic stirrer was 450 rpm. To imitate the temperature of the skin the receptor phase was thermostated at 32 °C. BGP-15 content was measured with mass spectrometry [23]. Release rate (*k*) of BGP-15 was determined from the slope of the amount of drug released per unit area versus the square root of time. Diffusion coefficient (*D*) was calculated from the amount of drug released per unit area (*Q*; μg/cm^2^), the initial drug concentration (*C*′_0_) and diffusion time (*t*) (Equation (1))

(1) $$D=\frac{Q^{2}\times \pi }{{\left(2[{C^{\prime }}_{0}]\right)}^{2}\times t}$$

Data were fitted to zero-order and first-order kinetics (Table 2).

To compare the release data of BGP-15 containing samples, similarity and difference factors were calculated, as a model independent approach: similarity, *f2* and difference, *f1* factor was calculated for each one [26].

(4) $$f_{1}=\frac{\sum_{j=1}^{n}\left|R_{j}-T_{j}\right|}{\sum_{j=1}^{n}R_{j}}\times 100$$

where *n* is the sampling number, *R_j_* and *T_j_* are the percent dissolved of the reference and the test products at each time point *j*, respectively.

(5) $$f_{2}=50\times \log\left\{\;{\left[1+\left(1/n\right)\overset{n}{\underset{j=1}{\sum }}w_{j}{\left|R_{j}-T_{j}\right|}^{2}\;\right]}^{-0.5}\times 100\;\right\}$$

where *w_j_* is an optional weight factor.

### 2.6. Biocompatibility Experiments

To evaluate cytotoxicity of BGP-15, the selected excipients and the formulated ointments, MTT assay was performed. The experiments were carried out on HaCaT cell line. HaCaT cells are human immortalized keratinocytes, thus they perfectly represent human skin. The cells were maintained by weekly passages in Dulbecco’s DMEM culture media. For MTT assay, the cells were seeded on a 96-well plate, at the density of 10,000 cells/well. When the cells fully grow over the well’s membrane the experiment is ready to perform. First we remove the culture media, then we apply the test solutions and incubate the cells with them for 30 min. After 30 min we remove the test substance and add MTT paint solution in 5 mg/mL concentration to the cells (tetrazolium bromide). Then we let it incubate with the cells for 3 h. The viable cells will transform the water-soluble tetrazolium bromide into formazan precipitate. When the incubation is completed, formazan precipitate is dissolved with the isopropanol–hydrochloride acid = 25:1 ratio. Then the absorbance of these solutions is measured by spectrophotometer (Fluostar Optima, BMG LABTECH, Offenburg, Germany) and it is directly proportional to the number of viable cells [27,28].

### 2.7. Inflammation Model Induced by Allyl-Isothiocyanate (AITC)

Animals were premedicated with BGP-15 before inducing inflammation. BGP-15 treatment happened by applying either ointments or solutions to mice ears. Both the solution and the ointments contained 10 *w*/*w*% BGP-15.

Anesthesia was induced by isoflurane (3–5%) with the help of a desktop anesthesia instrument, which made the process safe for the animals. Before each treatment and measurement mice were anesthetized. Inner and outer surface of mice’s left ear was smeared with 1% AITC dissolved in paraffin oil. This treatment happened 30 min later than the premedication with the ointments. After the ointments permeated into the skin (~30 min), AITC was applied and ear thickness was measured every 10 min [29,30,31].

### 2.8. Antioxidant Assay-Superoxide Dismutase (SOD) Assay

The antioxidant activity of BGP-15 was evaluated on HaCaT cell line, in different concentrations: 1; 5; 10; 50; 100 mg/mL. The solutions were prepared with PBS. As a negative control, PBS was selected. Cells were seeded on 24well plate with the cell density of 50,000/well and grown in CO_2_ incubator for 7 days at 37 °C. During the experiment, culture media was removed and the cells were incubated with the sample solutions for 1 h. For the experiment, artificial UVB radiation for 10 min was used to cause oxidative stress, induce free radical production after the treatment with BGP-15. Then, cells were collected with the help of a rubber policeman and centrifuged for 10 min, 1000 rpm, 4 °C. Cell pellet was homogenized in HEPES buffer and centrifuged again for 15 min, 10,000 rpm, 4 °C. Antioxidant activity of the supernatant was investigated with Cayman assay kit based on the instructions of the manufacturer. The experiments were performed in triplicate [32].

### 2.9. Total Antioxidant Capacity (TAC)

Twenty-weeks-old rats were assigned to the following three groups CMP3, CMP4, CMP5. First, rats were shaved, on the second day animals were treated with different ointments containing BGP-15 for 24 h. On the third day, the animals were treated on another skin region with ointments for one hour. After the treatment, animals were sacrificed with ketamine–xylazine overdose. Three skin samples were obtained from each animal, one from the area treated for 24 h, one from area treated for one hour and one from untreated area, which served as control. Total antioxidant capacity measurements were carried out with antioxidant assay kit (Sigma Aldrich, St. Louis, MO, USA) according to the manufacturer information. Briefly, approximately 100 mg of samples were homogenized with assay buffer, and centrifuged at 12,000 rpm for 15 min at 4 °C. The supernatants were used for the assay. The absorbance was measured at 405 nm using a MutiscanGo microplate spectrophotometer (ThermoFisher Scientific, OY, Ratastie, Finland). Values are expressed as a percentage of the value of the untreated region [33].

### 2.10. Luciferase Assay

Raw 264.7 cells stably transfected with the pNFĸB-Luc/neo. reporter construct were plated at 6 × 10^4^/well on luminescent assay plates in 200 μL of DMEM/F12 medium supplemented with 10% heat-inactivated FBS. After one-day culturing, the cells were treated with 100, 10 and 1 µM BGP-15 (dissolved with different emulsifiers (3 *w*/*w*%): SP50, SP70, PS750 in PBS) with or without LPS (100 ng/mL). After 6-h incubation (37 °C; 5% CO_2_), media was removed; cells were washed with 200 μL PBS/well and lysed with 20 μL Cell Culture Lysis Reagent/well for 10 min. After adding the firefly luciferase substrate (20 μL/well), luciferase activity was measured with Luminoscan Ascent Scanning Luminometer (Thermo Electron Corporation, Waltham, MA, USA).

Cell viability was routinely determined using trypan blue exclusion test during the assays to make sure that assays were always carried out on viable cells [34].

### 2.11. Statistical Analysis

Data were analyzed with GraphPad Prism 6 and presented as means ± SD. One-way ANOVA and Dunnett’s post hoc tests were performed to compare multiple groups and Unpaired *t*-test was chosen to compare two groups. Significant differences on the figures are signed with asterisks. Differences were regarded as significant, with *p* ˂ 0.05. All experiments were performed at least 3 times in quintuplicate.

## 3. Results

### 3.1. Texture Analyzing Studies

**Figure 2:**
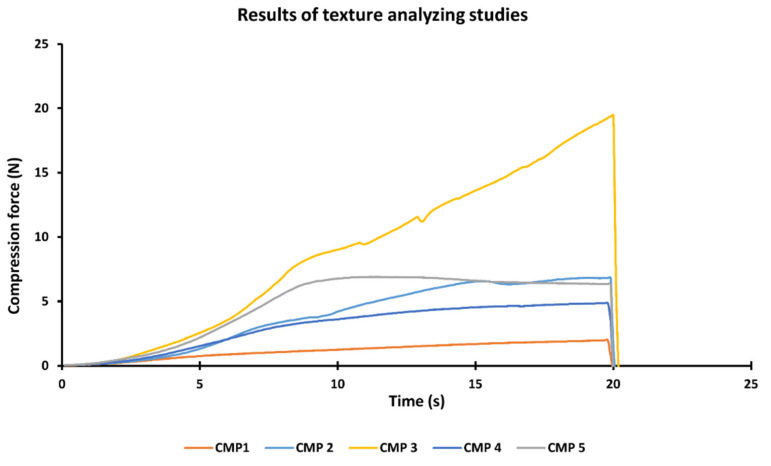
Resistance of the different ointments with the different surfactants. CMP 3 (made of SP50) showed the hardest consistency, while CMP 1 (made of Labrasol) showed the softest consistency. It is a representative measurement; the texture analyzer measures the values every 0.2 s. (Data present average values, *n* = 3).

The compression force required for the cylinder to intrude into the ointments is presented in Figure 2. Based on the compression force values, those formulations, which contain sucrose esters (CMP 3, 4, 5) have harder consistency. The formulation made of Labrasol (CMP 1) has the softest consistency, while the hardest is prepared with SP50 (CMP 3). Lower resistance values are preferable because of the easier applicability and better liberation of the active pharmaceutical ingredient.

### 3.2. In Vitro Release

**Figure 3:**
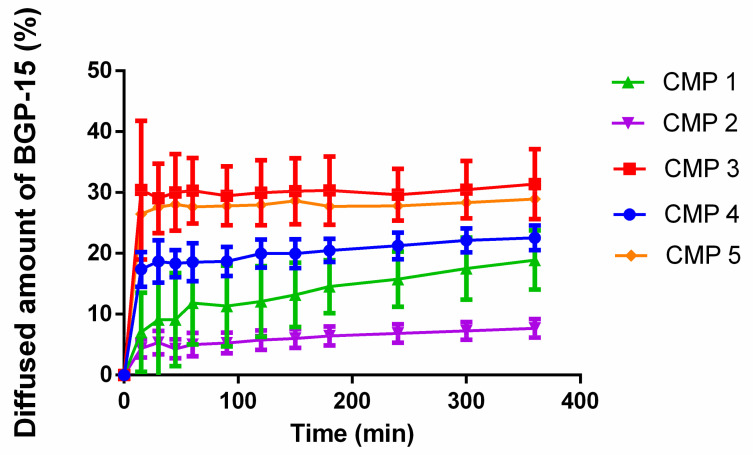
Release profiles of BGP-15 across cellulose acetate membrane from the different ointments. The best result belongs to CMP 3, while the lowest release values are produced by CMP 2. CMP 3 and 5 show similar results; in the case of CMP 1, a slower increasing tendency can be observed.

Figure 3 shows the diffused amount of BGP-15 of the different compositions across isopropyl myristate-impregnated cellulose acetate membrane. Those compositions, which contained sucrose esters produced better results. The best diffusion was achieved by CMP 3. The second best result was shown by CMP 5 and the lowest release rate was achieved by CMP 2. CMP 1 showed a slowly increasing tendency.

Release profiles of the different compositions are closer to zero-order kinetics than first-order kinetics (Table 3).

Our results showed that the best release rate was achieved by those formulations that contained sucrose esters. Release rates and diffusion coefficient values are listed in Table 4.

Release profiles of ointment compositions were compared to each other. The calculated difference and similarity factors are listed in Table 5. Two formulations are considered to be different if their difference factor (*f1*) is between 0–15 and similar if their similarity factor (*f2*) is between 50 and 100. Based on the calculated values, a great similarity is confirmed between CMP 3 and CMP 5. CMP 1 and 2; 1 and 4; 4 and 5 are considered to be similar as well.

### 3.3. MTT Assay

**Figure 4:**
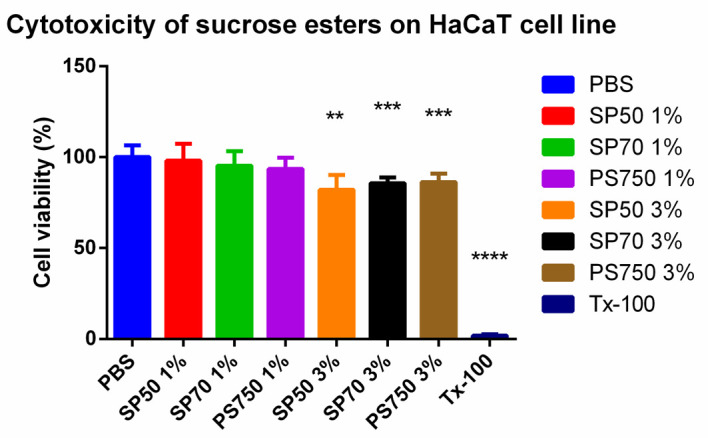
In vitro cytotoxicity of sucrose esters (SP50, SP70, PS750) in different concentrations on HaCaT cell line. Cell viability is determined as the percentage of PBS (negative control). All sucrose esters proved to be non-toxic since the cell viability is above 70% in each case. The data present the mean of 6 wells ± SD. For statistical analysis one-way ANOVA test and *t*-test were performed. Significant differences are marked with **, *** and **** indicate statistically significant differences at *p* < 0.01, *p* < 0.001, *p* < 0.0001.

The cytotoxicity of the ointments and excipients were tested on an HaCaT cell line. Cell viability values were compared to a positive control, which was Triton-X 100 and to a negative control, which was PBS. Sucrose esters were dissolved in PBS and they were tested in 1% and 3% concentrations. According to the results, SP50 in 1% concentration is the safest of all sucrose esters, but all of them proved to be safe and non-toxic in the investigated concentration range because the cell viability values are above 70% in each case. Results are demonstrated in Figure 4.

**Figure 5:**
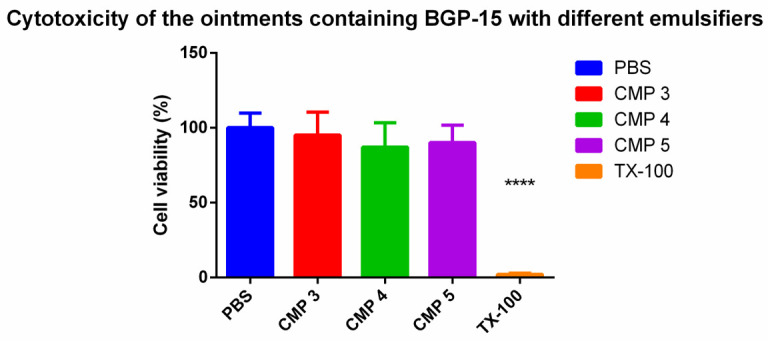
Cell viability assay on HaCaT cell line after incubation for 6 h with each composition. Cell viability is expressed as the percentage of PBS (negative control). Application of the incubated samples did not affect cell viability; the results are above 70% in each case. The data present the mean of 6 wells ± SD. For statistical analysis one-way ANOVA test and *t*-test were performed. Significant differences are marked with. **** indicates statistically significant difference at *p* < 0.0001.

An MTT assay of the ointments was performed with samples prepared by Franz diffusion chamber apparatus using pH = 5 buffer as the receptor phase. A total of 1 mL of samples were taken out of the receptor phase after 6 h of incubation. The results are presented in Figure 5. CMP 3 shows the best results, but all preparations seem to be non-toxic according to these results. Cell viability is over 70% in every case.

### 3.4. AITC Induced Inflammation Model

**Figure 6:**
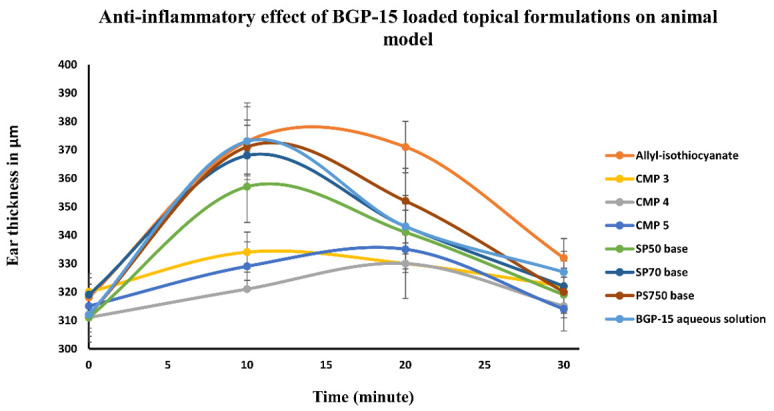
Changes in ear thickness as a result of different treatments with different formulations. CMP 4 showed the greatest decrease in the ear edema compared to the positive control. The formulations made without either BGP-15 or the penetration enhancers were not able to make such differences. Statistical analysis was performed with the help of one-way ANOVA test and *t*-test at 10th minute, where the inflammation is at the peak. Ointment compositions with BGP-15 have significantly decreased inflammation compared to the positive control (AITC).

As Figure 6 presents, all three ointments that contained BGP-15 significantly decreased ear thickness compared to the positive control (AITC by itself). CMP 4 (the ointment prepared with SP70 and BGP-15) shows the largest decrease of ear edema. Both CMP 3 and 5 (the ointments made of SP50 or PS750+ BGP-15) show similarly good results. Those preparations that did not contain the active ingredient (BGP-15) were not able to significantly reduce ear thickness, nor did the aqueous solution containing only BGP-15. This leads us to the conclusion, that BGP-15 has an anti-inflammatory effect but the aqueous solution could not penetrate into skin, while the ointments with the penetration enhancers were able to penetrate and prevent the inflammation.

**Figure 7:**
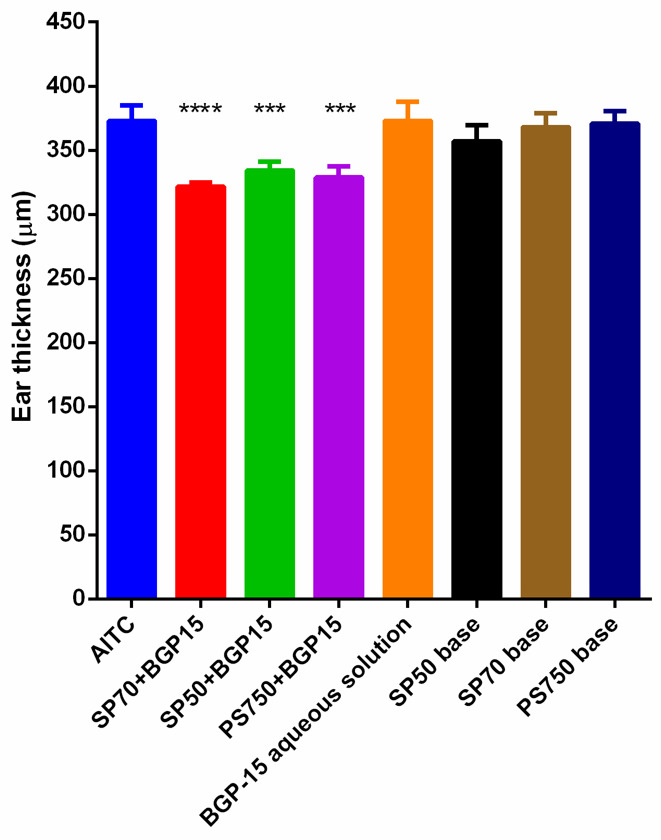
Ear thickness in the 10th minute of the treatment with the different compositions. Statistical analysis was performed with the help of one-way ANOVA test and T-test in the 10th minute, where the inflammation is at the peak. Ointment compositions containing BGP-15 have significantly decreased inflammation compared to the positive control (AITC). *** and **** indicate statistically significant differences at *p* < 0.001 and *p* < 0.0001.

While CMP 3, 4 and 5 (containing SP50 + BGP-15, SP70 + BGP-15, PS750 + BGP-15, respectively) reduced ear thickness significantly compared to AITC, the rest of the formulations did not result in a significant decrease in the thickness (Figure 7). Comparing the results of ear thickness of those formulations that contained the active substance and sucrose esters (CMP 3, 4 and 5) to the ointment bases (which did not contain BGP-15) with T-test, the results are significantly different (Table S1). The same applies to the comparison of BGP-15 aqueous solution to CMP 3 4 and 5. BGP-15 solution could not reduce ear edema, probably because it could not penetrate the skin; meanwhile, ointments meant a more suitable dosage form and guaranteed proper penetration and drug release.

### 3.5. Antioxidant Assay-Superoxide Dismutase (SOD) Assay

**Figure 8:**
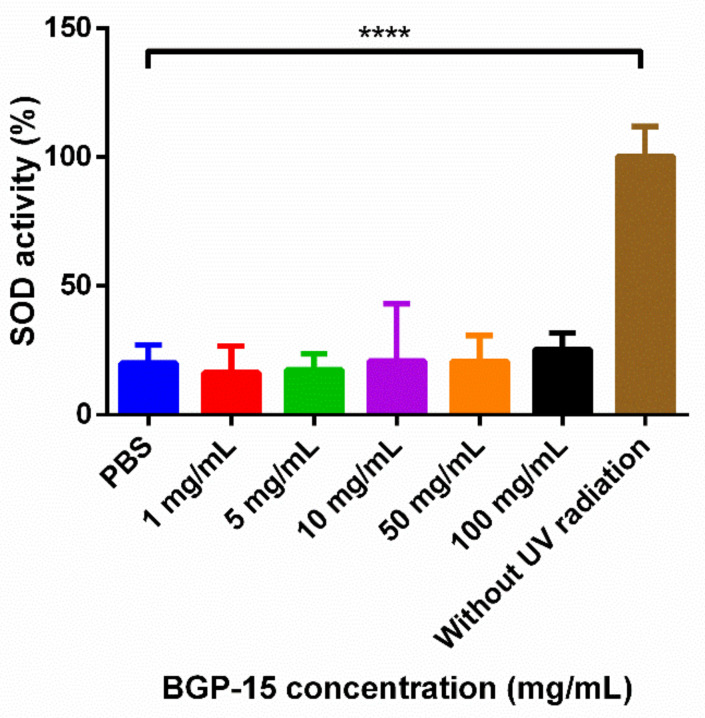
Effects of BGP-15 pretreatment on SOD enzyme level on HaCaT cells, exposed to UVB radiation. SOD enzyme activity is expressed as the percentage of SOD activity in HaCaT cells without UVB radiation. UVB exposed cells treated with PBS only serve as negative control. Data are expressed as the mean ± SD, *n* = 6. Comparison of the different groups happened with Dunnett’s multiple comparisons test. **** indicates statistically significant difference at *p* < 0.0001.

The function of an SOD enzyme is to protect cells from superoxide toxicity, which is one of the main reactive oxygen species (ROS) produced if cells are exposed to oxidative stress (e.g., UVB radiation). BGP-15 solutions, prepared with PBS in different concentrations, were selected for in vivo antioxidant assays. The SOD enzyme activity of the group that was not exposed to UVB radiation was taken as 100%. The SOD activity of the treated groups were compared to the enzyme activity of the group that was not exposed to UVB radiation [32]. Figure 8 shows that in the groups where the cells were previously treated with BGP-15 solution, SOD enzyme activity was decreased, similarly to the group that was treated with PBS only. The decreased level of SOD enzyme activity may be the consequence of the intense oxidative stress caused by UVB radiation, which resulted in severe cell damage. Pretreatment of the cells with any concentration of BGP-15 was not able to prevent the decrease in the SOD enzyme level.

### 3.6. Total Antioxidant Capacity

**Figure 9:**
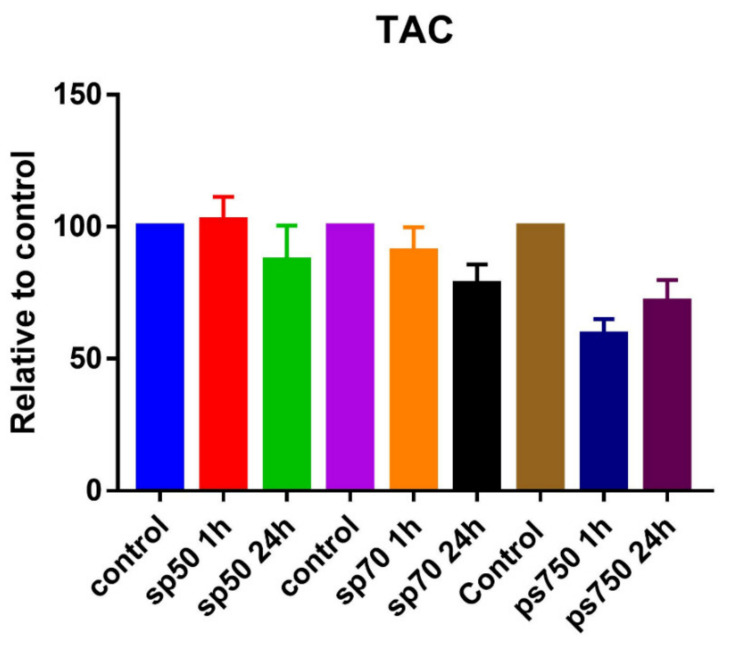
Effect of BGP-15-containing ointments on total antioxidant capacity. The results show no significant alteration with CMP 3 and 4, however, with CMP 5, TAC decreased.

The result of TAC measurement showed no significant alteration with CMP 3 (SP50) and CMP 4 (SP70); however, it decreased with CMP 5 (PS750) (Figure 9). Since no alterations were seen with the other two ointments, we speculate that it is probably due to the surfactant of the ointment. However, other assays such as the ORAC assay needs to be carried out to test the antioxidant capacity with other free radicals [35].

### 3.7. Luciferase Assay

**Figure 10:**
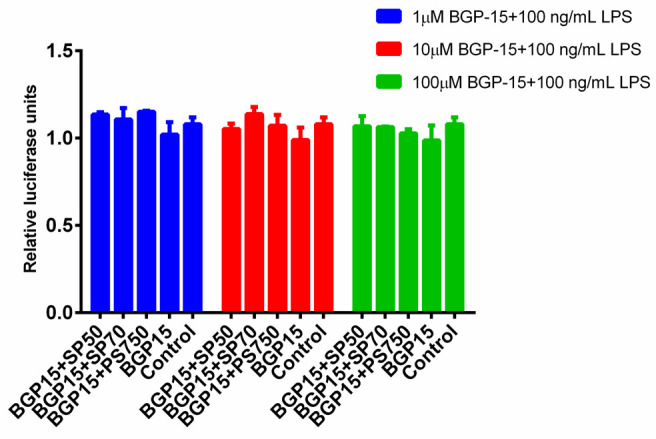
Effect of BGP-15 alone and together with surfactants (sucrose esters) on Raw264.7 cells induced with LPS. It appears that BGP-15 treatment was unable to reduce the LPS-activated macrophages’ NF-κB response.

The in vitro anti-inflammatory effect of BGP-15 was measured in mice macrophages through the response of NF-ĸB. During the experiment, the selected Raw264.7 cells were stably transfected with the pNFĸB-Luc/neo. reporter construct. The effect of BGP-15 on the NF-κB response of macrophages and the NF-κB of LPS-activated macrophages was investigated. With the experiment, we tried to verify if BGP-15 treatment could reduce the macrophage’s inflammatory factor (e.g., NF-κB) production. BGP-15 was applied in different concentrations (1, 10 and 100 µM). The measured luminescence was proportional to the activity of the transcription factor NF-κB. According to the results, it appears that BGP-15 treatment was unable to reduce the LPS-activated macrophages’ NF-κB response, (Figure 10) nor was it able to result in significant decrease in macrophage activity (Figure S1).

## 4. Discussion

BGP-15 is the subject of several researches due to its exceptional properties, however, current knowledge about the drug is still incomplete. The potential anti-inflammatory effect of BGP-15 is a frequently discussed hypothesis within different studies [36,37], but it is an under investigated research field, as is the topical application of the drug. Currently, BGP-15 creams are not available on the market despite having potential in the topical application of the drug in different indications (e.g., photo protection, inflammation) [12,36,37]. In the present study, different o/w emulsion ointments with different surfactants were formulated of BGP-15, and these compositions were investigated from different aspects, such as texture analysis and in vitro release. Texture analysis revealed that the compositions have adequate consistency; those formulations that contain sucrose esters have a slightly harder consistency. Based on the in vitro release tests, those three ointments (CMP 3, 4 and 5) that showed the best release profile were selected for further testing on animals. In parallel with these experiments, the biocompatibility studies of the ointments and the selected excipients were carried out on a HaCaT cell line. According to the MTT test results, all of the ointments and the excipients proved to be safe and non-toxic. The anti-inflammatory effect of BGP-15 ointments was investigated on C57BL/6J mice with an ear edema test. During this experiment, a local inflammation was induced on mice ears with AITC solution, and ear thickness was screened throughout the whole experiment. Mice ears were premedicated with the previously formulated ointments, or the BGP-15 aqueous solution. In the animal experiment, we found that those ointments containing the active ingredient significantly reduced or prevented the inflammation, while in those cases where mice were treated with those compositions that did not contain BGP-15, inflammation was induced almost to the same extent as when the ear was treated with the positive control (inflammatory agent (AITC)) alone.

Antioxidant properties, as a possible explanation of the anti-inflammatory effect of BGP-15, were evaluated with an SOD assay on a HaCaT cell line. Our results demonstrate that the drug was unable to protect the cells from UV radiation-caused oxidative stress; SOD enzyme levels decreased in exactly the same way as when the cells were only treated with PBS. TAC assay was also performed, but it showed no significant alteration with CMP 3 and CMP 4; however, it decreased with CMP 5, probably because of the surfactant’s chemical structure. Our findings are similar to the results of Sümegi et al., who tried to investigate the possible antioxidant effect of the drug by other methods, but they also came to the conclusion that the drug, although it inhibits mitochondrial ROS production, has no antioxidant effect [36].

Luciferase assay was performed as another possible way to explain the experienced anti-inflammatory effect of BGP-15. In the experiment, the in vitro anti-inflammatory effect of BGP-15 was measured in mice macrophages. Based on the results, it appears that BGP-15 treatment did not have any effect on LPS-activated macrophages’ NF-κB response, nor was it able to result a significant change in macrophage activity.

Our results can confirm the suggestions that BGP-15 has a protective effect against inflammation if applied topically, however, the exact mechanism of the effect remains unclear despite our attempts to identify it: we could only exclude a few possible explanations. In the scientific literature, a potential anti-inflammatory property is attributed to the PARP-inhibiting property of BGP-15. PARP inhibitors are able to protect cells from ROS-induced injuries. ROS production induces inflammatory processes [38,39]. Moreover, BGP-15 is able to protect mitochondria from ROS-induced damages by reducing mitochondrial ROS production [36]. BGP-15 is also able to decrease the expression of matrix metalloproteinase 9 (MMP9), which could be a possible explanation as well. Excess level of MMP9 leads to mitochondrial damage. BGP-15 blocks JNK as well, which is an inflammatory cytokine [40,41,42].

The composition of the ointments and the choice of appropriate penetration enhancers are important factors, not only in terms of pharmaceutical technology but also in terms of drug penetration and bioavailability. When the ointment compositions were determined, we tried to create formulations that contained the proper penetration enhancers and excipients to achieve the best possible bioavailability of BGP-15. Based on the scientific literature, the combination of sucrose esters (SP50, SP70, PS750) together with an active substance can enhance the effect, the same applies to Transcutol [19,43]. However, Cremophor and its derivatives do not always prove to be the best choice, Malingré et al. studied paclitaxel and they found that using Cremophor limited the absorption of the drug [44]. Our experience was similar, in the in vitro release test, CMP 2 (which contains Cremophor) showed the poorest results. Labrasol and IPM can be an advantageous combination as Okur et al. found that it can increase the permeation rate of naproxen in dermal drug delivery [45]. However, Labrasol does not always prove to be the right surfactant to choose either. Shafiq et al. investigated ramipril in their study and they came to the conclusion that Labrasol was not a suitable surfactant to enhance drug absorption [46]. In our case, the combination of sucrose esters and Transcutol proved to be the best choice according to the results of Franz diffusion, and these compositions achieved remarkable results in the animal experiment as well.

## 5. Conclusions

BGP-15 is a promising drug candidate with many beneficial effects, including its topical anti-inflammatory effect, which has not been investigated previously. In our study we tried to complete the current knowledge about the drug by studying a less investigated property of it. Our experimental work also highlights the importance of selecting the appropriate excipients as this affects the preparation from different aspects, both in advantageous and disadvantageous ways. Based on the results of our experiments, BGP-15 has an anti-inflammatory effect if applied topically, though the mechanism of the effect remains unclear. According to our results, BGP-15 has no antioxidant effect, neither can it decrease LPS-induced macrophage activation. O/W emulsion ointments were successfully formulated of BGP-15, the composition and the selected penetration enhancers affect the release of the active substance and, thus, the pharmacological effect of the preparation. Our results offer useful information for further future investigations about BGP-15 as its topical application is an under investigated research area.

## Figures and Tables

**Table 1:** Composition (CMP) of the formulated ointments.

Composition	CMP 1	CMP 2	CMP 3	CMP 4	CMP 5
Transcutol (1.4 g)	+	+	+	+	+
Emulsifier (3 g)					
Labrasol	+	−	−	−	−
Cremophor A6:A25	−	+	−	−	−
SP50	−	−	+	−	−
SP70	−	−	−	+	−
PS750	−	−	−	−	+
BGP-15 (10 g)	+	+	+	+	+
Cetostearyl alcohol (4.6 g)	+	+	+	+	+
Stearic acid (10 g)	+	+	+	+	+
Glycerol (5 g)	+	+	+	+	+
IPM (5 g)	+	+	+	+	+
Propylene glycol (5 g)	+	+	+	+	+
Purified water (ad 100.0 g)	+	+	+	+	+

**Table 2:** Mathematical model of drug release profiles.

Model	Equations [24,25]		Graphic
Zero-order	$Q_{t}=Q_{0}+k_{0}t$	(2)	The graphic of the drug-dissolved fraction versus time is linear.
First-order	$Q_{t}=Q_{0}\times e^{-k_{1}t}$	(3)	The graphic of the decimal logarithm of the released amount of drug versus time is linear.

*Q*_0_ is the initial amount of drug; *Q_t_* is the amount of drug remaining at time *t*; *Q_t_/Q_∞_* is the fraction of drug released at time *t*; *k*_0_ and *k*_1_ are the kinetic constants.

**Table 3:** Correlation coefficient values of the ointment compositions.

Kinetic Model		
Composition	Zero	First
CMP 1.	0.8549	0.8122
CMP 2.	0.7900	0.6249
CMP 3.	0.6465	0.1589
CMP 4.	0.7095	0.3765
CMP 5.	0.6502	0.1699

**Table 4:** Release rate and diffusion coefficient values of the ointment compositions.

Composition	Release Rate (*k*) (μg/cm^2^ × √min)	Diffusion Coefficient (D × 10^−4^; cm^2^/min)
CMP 1.	279.49	3.81
CMP 2.	123.39	0.972
CMP 3.	593.64	17.00
CMP 4.	402.12	6.48
CMP 5.	551.53	14.1

**Table 5:** Difference and similarity factors to compare the release profiles of the ointments.

Composition	*f1*	*f2*
CMP 4. vs. CMP 3.	34.19	49.06
CMP 4. vs. CMP 5.	28.98	54.19
CMP 4. vs. CMP 1.	35.59	56.54
CMP 4. vs. CMP 2.	70.49	42.66
CMP 5. vs. CMP 1.	54.26	40.49
CMP 1. vs. CMP 2.	54.18	56.47
CMP 5. vs. CMP 2.	79.04	32.78
CMP 3. vs. CMP 2.	80.58	30.71
CMP 3. vs. CMP 1.	57.61	37.62
CMP 3. vs. CMP 5.	7.33	80.75

## Data Availability

Data are available from the corresponding author with the permission of the head of the department. The data that support the findings of this study are available from the corresponding author (bacskay.ildiko@pharm.unideb.hu), upon reasonable request.

## Supplementary Materials

The following are available online at https://www.mdpi.com/article/10.3390/pharmaceutics13122037/s1, Table S1: Statistical analysis (*p* values and significance) by comparing the different formulations to each other. Figure S1: Effect of BGP-15 alone and together with surfactants (sucrose esters) on Raw 264.7 cells.
