# Supplementary material for: Nicotinic Amidoxime Derivate BGP-15, Topical Dosage Formulation and Anti-Inflammatory Effect
Source: Pharmaceutics. 2021 Nov 29;13(12):2037. doi: 10.3390/pharmaceutics13122037 (PMC8707203; doi:10.3390/pharmaceutics13122037)
Supplement: Supplementary file 1 [file pharmaceutics-13-02037-s001.zip › pharmaceutics-1423677-supplementary.pdf]

pharmaceutics

MDPI

Supplementary Materials: Nicotinic Amidoxime Derivate BGP-15, Topical Dosage Formulation and Anti-Inflammatory Effect

Ágota Pető, Dóra Kósa, Ádám Haimhoffer, Pálma Fehér, Zoltán Ujhelyi, Dávid Sinka, Ferenc Fenyvesi, Judit Váradi, Miklós Vecsernyés, Alexandra Gyöngyösi, István Lekli, Péter Szentesi,Annamária Marton, Imre Gombos, Barbara Dukic, László Vígh and Ildikó Bácskay

Table S1. Statistical analysis (*p* values and significance) by comparing the different formulations to each other.

Composition	*p* value	Significant	Asterisks
SP50 + BGP-15 vs. SP50 base	0.0076	Yes	**
SP70 + BGP-15 vs. SP70 base.	<0.0001	Yes	****
PS750 + BGP-15 vs. PS750 base	<0.0001	Yes	****
BGP-15 aqueous sol. vs. SP50 + BGP-15	0.0008	Yes	***
BGP-15 aqueous sol. vs. SP70 + BGP-15	<0.0001	Yes	****
BGP-15 aqueous sol. vs. PS750 + BGP-15	0.0005	Yes	***

, * and **** indicate statistically significant differences at $p < 0.01$, $p < 0.001$ and $p < 0.0001$, respectively.

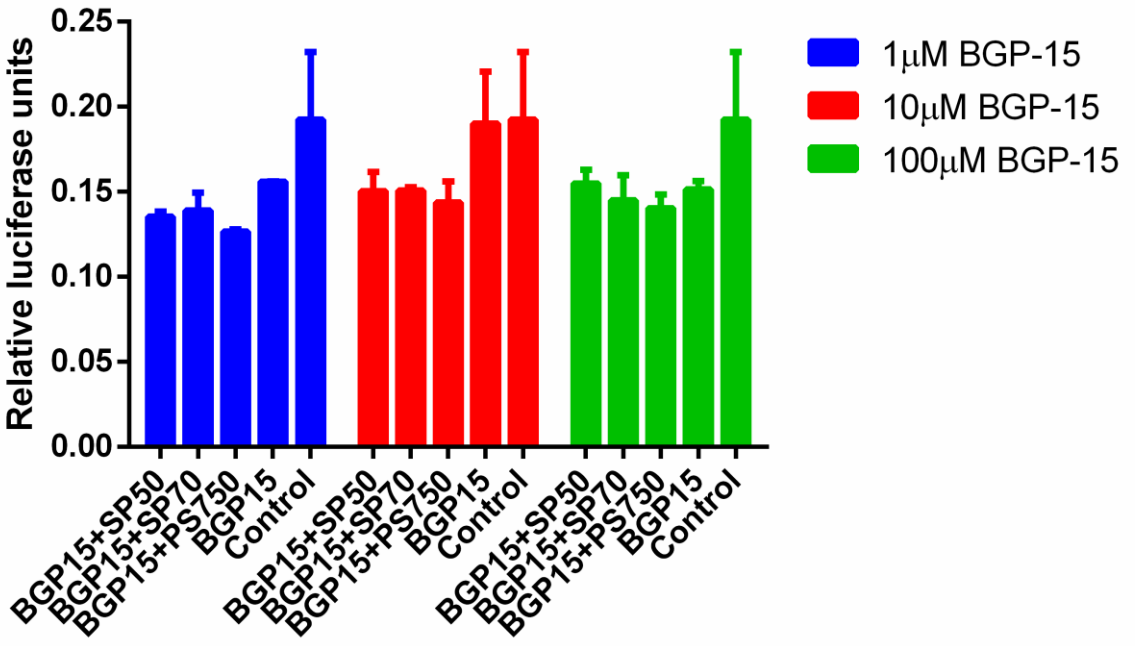

Figure S1. Effect of BGP-15 alone and together with surfactants (sucrose esters) on Raw264.7 cells. It appears that BGP-15 treatment resulted some sort of decrease in macrophage activity, but the results are not significantly different.
